# Systems biology approaches to understanding Epithelial Mesenchymal Transition (EMT) in mucosal remodeling and signaling in asthma

**DOI:** 10.1186/1939-4551-7-13

**Published:** 2014-06-02

**Authors:** Talha Ijaz, Konrad Pazdrak, Mridul Kalita, Rolf Konig, Sanjeev Choudhary, Bing Tian, Istvan Boldogh, Allan R Brasier

**Affiliations:** 1 Department of Biochemistry and Molecular Biology, The University of Texas Medical Branch, 301 University Blvd, Galveston 77555-1060, Texas, USA; 2 Sealy Center for Molecular Medicine, The University of Texas Medical Branch, 301 University Blvd, Galveston 77555-1060, Texas, USA; 3 Institute for Translational Sciences, The University of Texas Medical Branch, 301 University Blvd, Galveston 77555-1060, Texas, USA; 4 Department of Internal Medicine, The University of Texas Medical Branch, 301 University Blvd, Galveston 77555-1060, Texas, USA; 5 Department of Microbiology and Immunology, The University of Texas Medical Branch, 301 University Blvd, Galveston 77555-1060, Texas, USA

**Keywords:** EMT, Inflammation, NF-κB, TGFβ, Eosinophils, Systems biology, Innate immunity

## Abstract

A pathological hallmark of asthma is chronic injury and repair, producing dysfunction of the epithelial barrier function. In this setting, increased oxidative stress, growth factor- and cytokine stimulation, together with extracellular matrix contact produces transcriptional reprogramming of the epithelial cell. This process results in epithelial-mesenchymal transition (EMT), a cellular state associated with loss of epithelial polarity, expression of mesenchymal markers, enhanced mobility and extracellular matrix remodeling. As a result, the cellular biology of the EMT state produces characteristic changes seen in severe, refractory asthma: myofibroblast expansion, epithelial trans-differentiation and subepithelial fibrosis. EMT also induces profound changes in epithelial responsiveness that affects innate immune signaling that may have impact on the adaptive immune response and effectiveness of glucocorticoid therapy in severe asthma. We discuss how this complex phenotype is beginning to be understood using systems biology-level approaches through perturbations coupled with high throughput profiling and computational modeling. Understanding the distinct changes induced by EMT at the systems level may provide translational strategies to reverse the altered signaling and physiology of refractory asthma.

## 

Currently it is estimated that over 300 million adults and children suffer from asthma, representing a major public health problem worldwide. Over the past two decades, there has been a rise in asthma prevalence, such that about 8% of the population, more than 25 million Americans, were affected in 2010 [1]. Asthma is a heterogeneous disease whose presentation, clinical course, and response to therapy is determined by distinct pathobiological processes [2]. Two prominent asthma pathological processes are chronic inflammation and airway remodeling; these processes lead to the variable clinical manifestations characteristic of this syndrome [3]. Of these pathobiological processes, airway remodeling remains refractory to front-line glucocorticoid suppression therapy, and is associated with a subtype of asthma known as severe asthma. This subtype is associated with enhanced morbidity and accounts for disproportionate health care costs [4].

Recent exciting work has yielded insight into the dynamic and central role of the epithelium in asthmatic inflammation and remodeling. In this review, we discuss the cellular biology of an epithelial transcriptional reprogramming event known as epithelial-mesenchymal transition (EMT). Specifically, we examine the current knowledge of how growth factors and inflammation interface to produce EMT, of the role of innate immune cells in this process, and phenotypic modulation by the NF-κB signaling pathway. Due to the complexity of interacting signals and consequent global transcriptional reprogramming, systems-level studies are needed to fully understand critical pathways affected by EMT. The “systems” concept relies on global, experimental measurements of the genome, transcriptome, proteome, metabolome, and epigenome in response to external perturbation. One advantage of this approach is that it makes no *a priori* assumptions about the mechanisms underlying the response, allowing for the identification of new and less expected findings [5]. Systems biology has already provided new insights about the interaction between genes and the environment in asthma development [6], and environmental control of gene expression networks [7]. Here, we review findings of systems level perturbations and computational modeling that have shed light on how EMT produces dysregulation of the innate immune signaling pathway and we discuss how future system level studies will lead to potentially new translational interventions focused on modifying the reprogramming of the asthmatic epithelium.

## The epithelium is a central component of airway inflammation and remodeling

The airway mucosal barrier is produced by a relatively impermeable epithelial sheet connected by tight junctions that restrict fluid loss and limit inhaled particulate access to the internal milieu. The airway mucosa is a regionally diverse spectrum of highly differentiated epithelial cell types, each playing a specialized role in normal pulmonary function and host defense. For example, flattened simple squamous type I pneumocytes promote gas exchange, provide a barrier to minimize water loss, and prevent pathogens and toxins from access to the internal architecture; secretory goblet cells produce and secrete protective mucins into the airway lining fluid; ciliated epithelial cells produce protective epithelial lining fluid and mucociliary escalator for particulate clearance; and type II pneumocytes secrete surfactants responsible for maintaining alveolar patency [8]. In pseudostratified columnar epithelial tissue, basal epithelial cells serve a regenerative function, being responsible for transdifferentiation to repopulate ciliated epithelia, and Clara and goblet cell populations in response to injury or senescence [8].

Maintenance of epithelial integrity is critical to normal cellular signaling, pulmonary homeostasis, and response to toxicants and allergen exposures. Moreover, this dynamic and plastic cell type plays a key role in initiating innate signaling programs in response to physical, chemical and biological challenge through coordinating cytokine and defensin release, and secreting alarmins and Th2-differentiating cytokines [9]. Despite intense study of the Th2 polarization hypothesis [3,10], a body of evidence points to a disruption of the epithelial mucosal barrier and its chronic regenerative process as playing a key pathogenic role in diverse forms of asthma.

Asthma is disease driven, in part, by epithelial injury and repair. Representing the principal cell type between the environment and internal milieu, the epithelial cell not only plays a critical role in the activation and coordination of the innate immune response, but also in tolerance and control of airway hyper-reactivity. It is well established that increased epithelial cell fragility with attendant denudation/shedding of epithelial cells and the consequent disruption of its barrier function enhances allergic sensitization [8]. CC chemokines CCL2, CCL20 and IL-12p40 produced by stimulated epithelial cells activate tissue-resident dendritic cells to produce Th2 polarization characteristic of asthma [3,11]. The loss of apical polarity, increased Goblet cell number (metaplasia) and expansion of the myofibroblast population are characteristic histological features of severe asthma. In animal studies, cellular lineage experiments have shown that epithelial cells contribute significantly to the myofibroblast population [12]. These data suggest that the epithelium plays significant pathogenic roles in the genesis and maintenance of reactive airway disease [9].

## Inducible epithelial phenotypes: Epithelial-Mesenchymal Transition (EMT) and transdifferentiation

Under normal conditions, the airway epithelial cells signal to an attenuated sheath of subepithelial mesenchymal cells, forming an epithelial-mesenchymal unit (EMU). Integrity of the EMU depends on growth factors secreted from epithelial cells; growth factor secretion is rapidly increased in response to epithelial injury to promote regeneration of this critical mucosal surface [13]. Injury, subsequent inflammation and the loss of epithelial basement membrane integrity also promote epithelial cell activation by extracellular matrix-associated factors. These resident factors include the growth factors epidermal growth factor (EGF), fibroblast growth factor (FGF), and TGFβ, whose actions are modified by innate immune cytokines (such as IL-6, IL-1β, MCP-1 and RANTES) produced by epithelial and tissue resident leukocytes [9,14]. These factors transform specialized epithelial cells to become motile, fibroblast-like cells; this process is referred to as type II EMT [15], a response central to repair after tissue injury. In addition to TGFβ, hypoxia is a strong inducer of EMT in kidney and lung epithelia [16,17]. Reactive oxygen species (ROS) produced in hypoxic settings are as potent inducer of EMT as that of TGFβ [16,18]. EMT can also be initiated by other growth factors such as FGF-2, EGF, and connective tissue growth factor (CTGF) [19].

**Figure 1:**
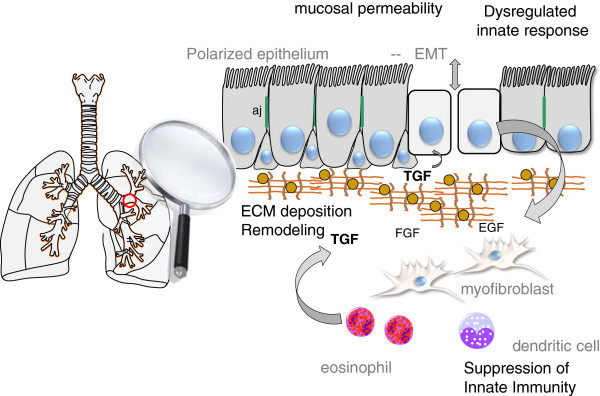
**Schematic view of EMT in the airway.** Shown is a segment of airway epithelium and homeostatic properties affected by type II EMT. The induction of type II EMT is modulated by growth factors released from the ECM and tissue resident leukocytes (eosinophils) and results in loss of adherens junctions (aj), loss of apical-basal polarity and disruption of mucosal barrier function, enhanced ECM deposition and dysregulated innate signaling. Also, there is suppression of innate immunity in some asthmatics but the link between EMT and suppression of innate immunity needs to be explored further. TGFβ, transforming growth factor beta; FGF, fibroblast growth factor; EGF, epithelial growth factor.

Because TGFβ is the most potent and most well described inducer of EMT, we will discuss its role in modifying the airway epithelia in detail. In the airway mucosa, TGFβ induced type II EMT leads to disruption of mucosal barrier function by inducing the loss of apical polarity, reduced epithelial cadherin (ECad) and disruption of epithelial adherens junctions (Figure 1; [9,10]). In addition, type II EMT enables transformed epithelial cells to express α-SMA stress fibers and intermediate filament vimentin, to produce ECM through secretion of collagen and fibronectin, and to increase expression of matrix metalloproteinases (MMPs) to promote airway remodeling. Finally, EMT also produces complex alterations in the innate immune response [20], a point discussed in detail below. Although type II EMT may be a protective mechanism for tissue repair, excessive and prolonged EMT processes can lead to fibrosis and organ damage as observed in lung [12], liver [21] and kidney fibrosis [22].

Other injury-induced epithelial differentiation paradigms may represent “formes frustes” of EMT. Chronic stimulation with growth factors EGF and amphiregulin, modified by Th2-derived IL-4 and IL-13 cytokines are thought to be responsible for expansion of the mucus-producing Goblet cell population [9]. Goblet cell hyperplasia is the product of trans-differentiation of ciliated and Clara cells. Goblet cells are responsible for expression of MUC5A and MUC5B, glycoconjugates that alter the hydro-elastic properties of mucus in asthma. In this way, stressed epithelial cells within the inflammatory milieu of airways disease are induced to undergo distinct phenotypic switches in ways that fundamentally alter the normal function of the epithelium and play an important role in airway remodeling in asthma.

## EMT in asthma

In asthmatic patients, the airway is fragile with decreased cell-to-cell adhesion, a process due, in part, to reduced ECad expression [23]. Immunoreactivity for TGFβ and its activated intracellular pathway protein, phospho-Smad2 (p-Smad2), are increased in mucosal biopsies suggesting the presence of active TGFβ signaling in asthmatic epithelium [24-26]. These and other lines of evidence suggest that EMT plays an important role in pathogenesis of subtypes of asthma [14,27]. Isolated human primary airway epithelial cells from asthmatics lose zona occludin-1 (ZO-1) expression at lower TGFβ concentrations than that required for normal epithelial cells [28]. In studies of pseudostratified columnar epithelial cells cultured in an air-liquid interface, TGFβ-induced EMT occurs in the basal cell layer, a stem-cell like population involved in trans-differentiation of numerous epithelial cell types [28]. Evidence of detrimental effects of TGFβ in the lung comes from mouse models where instillation of TGFβ1 into lungs or ectopic expression in the airway epithelium using adenovirus vectors induces airway collagen mRNA and protein deposition [29]. Consequently, neutralization of TGFβ reduces pulmonary fibrosis, collagen deposition and smooth muscle proliferation [30]. Accumulation of TGFβ in the bronchoalveolar lavage fluid of chronic asthma patients as well as in sensitized experimental animals challenged with OVA suggests a critical role of TGFβ in airway remodeling and lung EMT [28,31,32]. These data suggest that there is dysregulated TGFβ signaling in asthmatics and allergic airways which leads to disruption of the epithelial barrier function, mucosal remodeling and fibrosis.

## Signaling pathways producing EMT

**Figure 2:**
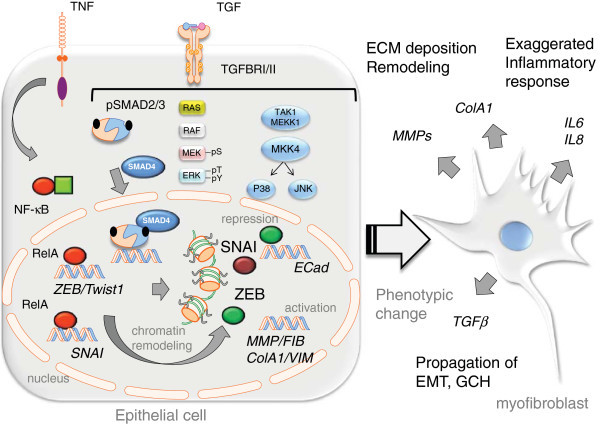
**Pathways activated by TGFβ to induce EMT.** An idealilzed epithelial cell before and after EMT showing the canonical (smad) and non-canonical TGFβ intracellular signaling pathways. The TGFβ signaling is upstream of the master transcriptional regulators, snail (SNAI1/2), zebra (ZEB) and Twist (Twst) that induce transcriptional reprogramming and chromatin remodeling through histone modification. Stimulation of TGFβ pathways result in repression of ECad (*CADH1*) and upregulation of fibronectin (*FIB*), collagen 1 (*Col1*) and α-SMA in the epithelial cell. Known interconnections with the TNF-NFκB inflammatory response are diagrammed.

The molecular signaling of how TGFβ1 initiates EMT has been studied in some detail (Figure 2). Free TGFβ binds to TGFβ receptor type II (TGFβRII), a transmembrane serine, threonine kinase that recruits and phosphorylates TGFβRI. In the canonical pathway, the activated TGFβRI/II complex then recruits Smads 2/3, cytoplasmic transcription factors that are, in turn, phosphorylated by TGFβRI on serine residues. Phosphorylated Smad2/3 binds to Smad4 and the complex then translocates to the nucleus. The activated Smad 2/3/4 trimer binds to Smad-binding elements in the regulatory regions of junB and c-Jun, and modulates transcription with other coactivators including the cAMP-response element binding protein (CBP)/p300 histone acetyltransferases [33].

TGFβ1 binding to its receptors also stimulates noncanonical signaling pathways including PI3K/Akt, Ras small GTPases, Wnt/β-catenin, ERK, p38, and JNK. Although Smad and non-Smad pathways are both activated by TGFβ, it is difficult to distinctly separate the actions of Smads from non-Smad proteins since there is significant cross-talk between the signaling arms with Smads regulating some non-canonical pathways and *vice versa*[34]. In most instances, Smad-dependant signaling is dominant in mediating EMT but non-Smad proteins are required to complete the cellular transformation.

Activation of all three MAPKs leads to cell proliferation, but production of fibronectin is dependent on ERK1/2 and JNK [35]. Mechanistically, it has been demonstrated in a fibrosarcoma cell line that TGFβ1 activates MAPK4-JNK1, leading to dimerization of the c-Jun and ATF2 transcription factor to initiate transcription of the fibronectin gene [36]. Recent findings in Non-Small Cell Lung Cancer cell lines indicate that TGFβ1 induced phospho-Erk1/2 mediates a decrease in ECad and an increase in fibronectin expression [37]. Inhibition of JNK, p38 and Akt activities without affecting Smad phosphorylation blocks TGFβ1 induced α-SMA, SNAI1 and collagen I in primary alveolar epithelial cells [38]. Another important non-Smad pathway linking epithelial tight junctions to cytoskeletal changes is the β-catenin pathway. TGFβ1 stimulation causes an increase in α-SMA via SMAD3-β-catenin-CBP interaction on the α-SMA promoter [39]. β-catenin is part of the complex that mediates binding of ECad to the actin cytoskeleton. Therefore, downregulation of ECad allows for more availability of β-catenin to bind to Smads, thus providing a unifying mechanism by which epithelial cells lose their apical polarity and undergo cytoskeleton rearrangement to become motile myofibroblasts.

Currently, it is thought that three families of transcription factors function as key regulators of EMT: snail (SNAI)1/2, zebra (ZEB)1/2, and Twist 1/2 families [40]. In the canonical pathway, TGFβ-stimulation upregulates SNAI expression via Smad3/4. A SNAI1-smad3/4 complex then binds to regulatory promoter regions of ECad and ZO-1, leading to their repression [41]. Smad signaling also increases ZEB1/2, a transcriptional repressor of the miR-200 family of micro RNAs. The mir-200 family represses translation of ZEB1/2, TGFβRI and Smad2. In this manner, miR-200 repression by ZEB1/2 promotes EMT by increasing TGBR mediated signaling [42]. In addition, these transcription factors control the expression of matrix metalloproteinases (MMPs) and ECM proteins such as collagens [40].

## Modulators of the EMT program

Several studies suggest that the ability of TGFβ to induce EMT is modulated by innate cytokine stimulation, morphogen signaling, allergen exposure and through ECM interactions. Studies of transformed alveolar basal epithelial cells show that TGFβ-induced EMT is accelerated by the presence of members of the proinflammatory TNF/ IL-1 superfamily of cytokines [43-45]. TNF/ IL-1 through intracellular adapters trigger the innate immune response activating downstream Ras GTPase, p38 MAPK and JNK, key components of the noncanonical TGFβ signaling pathway (Figure 2). Additionally, IL-1/TNF controls NF-κB, a master regulator of airway inflammation [46] that regulates a ~4,000 member gene network mediating anti-apoptosis, inflammation, and adaptive immunity [47,48]. NF-κB has been identified as a key regulator of the core EMT program, deserving specific mention. In studies of cancer-associated (so-called type III) EMT, NF-κB is required for growth factor-induced SNAI expression by directly inducing expression of the SNAI gene [49]. Similarly, NF-κB upregulates the ZEB1/2 transcription factors that mediate transcriptional silencing of the ECad gene and upregulation of the vimentin (VIM) promoter. In a similar manner, TNF-inducible Twist1 expression is mediated by NF-κB [50], explaining, in part, how the IL-1/TNF superfamily of cytokines mediates EMT. Other exciting studies have shown that activated NF-κB may also directly bind and stabilize SNAI, promoting its pro-EMT activity [51]. In addition to the well described TNF/IL-1 ligands, the superfamily member LIGHT (TNFSF14) is a T cell coactivator that mediates chronic airway inflammation and is associated with asthma disease severity [52]. LIGHT modulates TGF-β1-induced EMT by suppressing E-cadherin and enhancing vimentin expression [53]. Interestingly, LIGHT can also induce EMT independently from that of the TGF-β1 pathway through activation of Erk1/2 signaling [53]. Together these data indicate that TNF/IL-1 superfamily through NF-κB signaling plays a central, upstream coordinating role in EMT through both transcriptional and post-transcriptional actions.

The actions of TGFβ on EMT are further controlled by the Notch pathway, a highly conserved morphen control pathway involved in epithelial intercellular communication and cell fate specification. In cultured rat alveolar epithelial cells, Notch activation increases the expression of α-SMA, collagen I and vimentin and decreases expression of epithelial markers. Notch promotes EMT in airway epithelia by increasing expression of TGFβ ligands and inducing myofibroblast differentiation of alveolar epithelial cells via the TGFβ -Smad3 pathway [54].

Vascular endothelial growth factor (VEGF), matrix metalloproteinase-9 (MMP-9), and Th2-type cytokines (IL-5, -13, -4 and -9) produced by adaptive immune cells in the lung have also been observed to modulate EMT [55-58]. Moreover, the pro-inflammatory IL-17A cytokine directly regulates EMT and collagen synthesis in a TGFβ1-dependent process [59]. Neutralizing IL-17A promotes autophagy and attenuates pulmonary fibrosis [59]. This latter mechanism may be important in the pathogenesis of neutrophilic asthma, a subtype of severe steroid refractory asthma characterized by Th17-mediated neutrophilic inflammation.

The ECM is an interlocking mesh of collagens, elastic fibers and glycoproteins that plays a critical role in epithelial polarization, proliferation and signaling [60]. Dynamic remodeling of the ECM in response to injury releases local depots of TGF to stimulate proliferation, repair and EMT. In addition, the composition and rigidity of the ECM is another important modulator of epithelial cell signaling and function. Exposure of primary alveolar cells to fibronectin or fibrin led to robust EMT, a phenomenon dependent on αVβ6 integrin [12]. In a follow-up study, deletion of α3 integrin in lung epithelium prevented mice from developing fibrosis and led to a decrease in myofibroblast population and collagen I expression after bleomycin injury [61]. These findings strongly suggest that EMT is regulated by contextual factors such as cytokines and ECM interactions through integrin signaling.

Finally, extrinsic environmental stimuli can induce EMT. One prominent example is the house dust mite aero-allergen; exposure to house dust mite potentiates TGFβ-induced EMT of airway epithelial cells by stimulating myosin light chain phosphorylation and actin reorganization and enhanced β-catenin signaling [62]. This is mechanistically important because the β-catenin-Wnt pathway regulates expression of SNAI2/Slug a transcriptional repressor of ECad. Infection with Respiratory Syncytial Virus (RSV), a paramyxovirus widely considered a risk factor for development of allergic asthma later in life, is also characterized by increased expression of SNAI1 and TGFβ1, master regulators of EMT [63].

## Effector cells of airway remodelling and EMT; contribution to potential endotypes of asthma

Airway remodeling, in addition to inflammation, abnormal neurogenic and contractile response, has recently been appreciated as one of the contributors to the symptoms, abnormal physiology and natural history of asthma and therefore is likely to generate manifestation of the disorder as phenotype. This novel concept led to a series of studies delineating cellular and molecular mechanisms of airway remodeling and clinical trials aiming to identify subpopulations of asthmatics with similar disease mechanism (endotype) [64,65]. Airway remodeling is a collective term for structural alterations of airways encompassing the subepithelial fibrosis, myofibroblast hyperplasia and smooth muscle hypertrophy. The role of EMT in the development of subepthelial fibrosis, has been considered [28,66] upon observation of elevated TGFβ1 production by eosinophils and fibroblasts in patients with severe and moderate asthma [67,68]. Studies of allergen-induced airway remodeling in transgenic mice suggested an important role of TGFβ1, VEGF, Th2 cytokines (IL-5, IL-4, IL-13), and epithelial derived NF-κB regulated chemokines in airway remodeling, while eosinophils have received attention as cells contributing to thickening of reticular basal membrane [14]. Mechanistically, eosinophils are the source of potent cytotoxic mediators, such as leukotrienes, metalloproteinases, and growth factors, including TGFβ1 [69]. In patients with severe asthma, eosinophils constitute the majority of TGFβ1 producing cells as bronchial biopsies showed that 65% of TGFβ1 mRNA-positive cells are eosinophils and 75% of lung eosinophils were positive for TGFβ1 mRNA [70]. Animal studies of airway inflammation using interleukin-5 and eotaxin-transgenic models indicate that goblet cell hyperplasia, epithelial hypertrophy, and focal collagen deposition are eosinophil-dependent [71,72]. Complementing these observations, ΔdblGATA eosinophil-deficient and IL-5-deficient mice showed significant reduction in subepithelial collagen deposition and smooth muscle proliferation upon single challenge or chronic exposure to allergen [56,73]. Furthermore, allergen challenge experiments in IL-5-deficient mouse showed decreased numbers of TGFβ1 -positive cells in the peribronchial region and reduced expression of TGFβ1 in the whole lung [57]. The role of eosinophils in mediating EMT was directly shown in experiments where intratracheal instillation of bone marrow-derived eosinophils into mouse airways resulted in a marked deposition of type I collagen and significant fibrosis [74]. These changes were accompanied by decreased ECad and increased α-SMA expression 21 d after eosinophil instillation. These findings complemented an earlier study employing anti-IL-5 antibody in patients with severe eosinophilic asthma refractory to corticosteroids that demonstrated that a reduction in eosinophils was associated with decreased basement membrane deposition of tenascin, lumican and procollagen type III [75].

However, there are several observations suggesting that airway remodeling in asthma may occur without significant involvement of eosinophils [76] or even in the absence of inflammation. For example, repeated methacholine-induced bronchoconstriction in mild atopic asthmatics resulted in airway remodeling comparable to that of induced by allergen challenge [77]. This remodeling was accompanied by increased expression of TGFβ1 in the bronchial epithelium without airway eosinophilia suggesting heterogeneous upstream mechanisms mediate airway remodeling. Besides eosinophils, macrophages of alternative phenotype (known as the “M2 type”) were shown to participate in lung fibrotic processes in experimental models of nematode infection in IL-4R deficient mice [78,79]. Macrophage polarization toward the M2 phenotype is characterized by increased expression and secretion of TGFβ1 and can be achieved by stimulation of macrophages with IL-4, IL-13 [80] and glucocorticoids [81]. Although the ability of M2 macrophages to produce TGFβ1 upon stimulation of cytokines overexpressed in asthmatic airways implied their involvement in fibrotic and remodeling processes in asthma, recent observations have, however, shown overlapping of M1/M2 phenotypes and conversion of one phenotype to another depending upon the inflammatory response [82]. Similarly to macrophages, bronchial smooth muscle cells and dendritic cells were considered to play role airway remodeling after observation of enhanced secretion of TGFβ1 in response to neutrophil-derived elastase [83,84]. Since neutrophils infiltrate airways in severe and chronic asthma, their ability to mediate production of TGFβ1 may affect airway remodeling seen in this group of patients [85].

The correlation of airway remodeling with features of EMT, expression of TGFβ1, severity of disease, resistance to glucocorticoid therapy and airway eosinophilia or neutrophilia can be used to define distinct molecular phenotypes of asthma and to some degree asthma endotype. The asthma “phenotype” is the result of a complex constellation of pathophysiological processes, whereas an “endotype” represents a subtype of disease defined functionally and pathologically by a singular molecular mechanism translating into a treatment response [86,87]. Although no single endotype of asthma has yet been fully characterized, several potential endotypes were proposed based on clinical characteristics, biomarkers, histology and treatment response. In this regard, the phenotype of steroid-refractory asthma may include the potential endotype of steroid-insensitive eosinophilic asthma, a relatively rare form of asthma with airway eosinophilia, histological features of airway remodeling, glucocorticoid resistance and sensitivity to anti-IL-5 treatment [86]. This endotype mechanistically resembles IL-5 transgenic or IL-5 deficient mouse models [75].

Future research exploring the role of EMT in airway remodeling may delineate that EMT may play a role common to several endotypes. For example, EMT may contribute to steroid insensitivity as suggested by decreased induction of anti-inflammatory genes by glucocorticoids in TGFβ1-EMT transformed cells A549 cells [88]. In neutrophilic asthma, another subtype of severe steroid refractory asthma characterized by Th17-mediated neutrophilic inflammation and the absence of airway eosinophilia, airway remodeling may result from EMT induced by TGFβ1 released from elastase-stimulated bronchial smooth muscle cells and/or dendritic cells [85]. Furthermore, even the endotype of Th2-high, early-onset asthma with high eosinophilia and good response to steroid treatment exhibits some features of airway remodeling [89] as shown by thickening of reticular basement membrane by median age of 29 months in preschoolers with recurrent wheezing [90]. Although not yet defined, this endotype, driven by IL-4 and IL-13, may feature a role of TGFβ1 released from M2 polarized macrophages or from macrophages activated during phagocytosis of apoptotic eosinophils. Similarly, the severe, late-onset eosinophilic asthma endotype, representing 20% of the severe asthma population [91], also shows features of TGFβ1 mediated EMT and airway remodeling in spite of good response to systemic corticosteroid therapy.

In conclusion, future studies aiming at defining asthma endotypes should consider identification of mechanisms and respective biomarkers of TGFβ1 mediated EMT. Search for specific endotype biomarkers must focus on homogenous group of patients according to underlying disease mechanisms described by histological features (e.g. eosinophilia, M2 polarized macrophages), cytokine signatures (e.g. TGFβ1, IL4, IL-5) and responsiveness to anti-cytokine and glucocorticoid therapy. Systemic studies need to be directed at the cellular, molecular, and genetic factors that are responsible for determining why only some asthmatics develop significant remodeling. The information gathered from these studies will impact strategies employed to combat this increasingly complex and heterogeneous disorder.

## Effects of EMT on epithelial innate signaling responses

**Figure 3:**
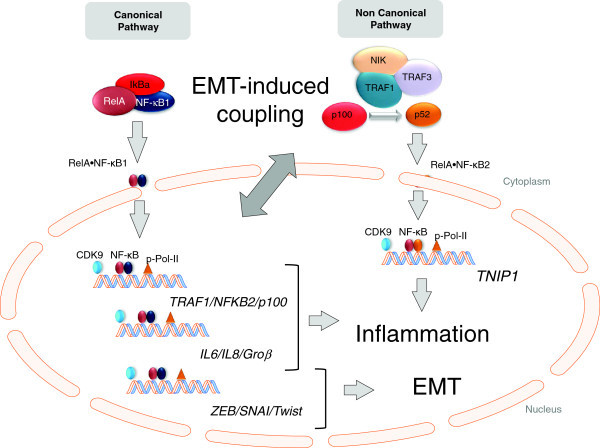
**Consequences of EMT on innate signaling.** The innate NFkB pathway is composed of the rapidly responding (canonical)- and the slower responding (noncanonical) pathways. The canonical pathway controls the inflammatory response, and is coupled to the noncanonical pathway through a feed-forward pathway by the synthesis of TRAF1 and NFκB2, two rate limiting factors in noncanonical pathway activation. Through epigenetic reprogramming and enhanced transcriptional elongation through the cyclin dependent kinase (CDK), the rate-limiting TRAF1/NFκB2 translation is reduced, resulting in enhanced pathway activation in EMT.

In addition to affecting glucocorticoid response discussed above, changes in the phenotype of epithelial cells in asthma may determine effects on innate signaling responses [20]. Our earlier systems-level study observed that EMT had dramatic effects on the induction of the innate NF-κB pathway by producing an exaggerated inflammatory response and by reducing the coupling interval between the rapid canonical response and the slower noncanonical pathway. Previous work has shown that type III EMT is associated with epigenetic modifications on specific genetic loci, including induction of the euchromatin mark H3-lys4 trimethylation, or the transcriptional mark H3K36 trimethylation [20,92]. Our studies in primary human airway cells indicated that enhanced responsiveness of the NF-κB pathway is mediated by accumulation of H3K4 trimethylation marks in NF-κB-dependent genes working in concert with transcriptional elongation [20]. Transcriptional elongation is a characteristic gene control mechanism of innate response genes mediated by the cyclin-dependent kinase (CDK9). Our findings indicate that EMT induces genetic reprogramming of innate response genes through repositioning of activated histone transcriptional marks and through modification of CDK activity (schematically diagrammed in Figure 3). As a result, the dynamic inflammatory response of the epithelial cell is fundamentally changed by the EMT state. However, the mechanism by which specific loci are targeted for chromatin modifications is still unclear.

Further systems level studies will help to understand the complexity of the epigenetic reprogramming with the inducible phenotype produced by EMT. These approaches include mathematical modeling of signaling pathways to infer how EMT influences dynamic responses, systematic phosphoprotein profiling in response to cellular stimulation to identify coupling of intracellular signaling pathways and computational prediction of transcription factor binding sites at a genome-wide level. Application of this latter methodology has identified motifs that likely regulate dynamics of Polycomb-mediated histone modifications during murine stem cell differentiation [93]. Furthermore, whole genome studies have shown H3K36me3 is a specific epigenetic signature that modulates selection of exon-intron junctions, thereby allowing expression of mRNA splice variants characteristic of EMT [94,95].

## Effects of EMT on innate/adaptive immune cell signaling

Severe asthma is associated with defects in innate immunity [4], including increased susceptibility to rhinovirus (RV) infections, leading to increased lower respiratory tract inflammation and hyper-reactivity. EMT-induced remodeling of the epithelial basement membrane promotes sensitization to inhaled allergen by causing persistent dendritic cell (DC) activation and migration [96]. Two studies have shown that sentinel respiratory cells replicate rhinovirus more efficiently and show deficient induction of type I/III IFNs, IFNs-β and -λ, with impaired ability to induce apoptosis [97,98]. This defect in type III IFN production was highly correlated with severity of RV-induced asthma exacerbation and virus load in experimentally infected human volunteers, suggesting that asthmatics have an acquired defect in type III IFN production in sentinel cells of the airway. The contributions of EMT in modulating the innate and adaptive immune responses, such as IFN-γ and TGF-β production, require further exploration.

Although the effect of type II EMT on innate and adaptive immunity in the airway is incompletely delineated, the understanding of type III EMT on adaptive immunity has been extensively investigated. Epigenetic modifications induced in type III EMT suppress transcription of the NK cell activating receptor, NKG2D [99], secretion of soluble decoy ligands MICA/B [100] or by localized cell-surface expression of NKG2D ligands [101]. SNAI1, the EMT master regulator, promotes immunosuppression by inducing differentiation of immature DCs into regulatory DCs with low MHC class II expression and associated costimulatory molecules and prevents NKG2D expression in NK cells [102], thus suppressing the innate immune response. Tumor cells also acquire resistance to lysis by antigen-specific CTLs following EMT by induction of autophagy through Beclin 1 [103]. Even though there is overlap between type II and type III EMT signaling and EMT’s effect on immunity, more work is required to determine which features of EMT are shared by the two EMT programs and to understand the role of EMT in modifying the innate immune response, autophagy and immunoediting in asthma.

## Systems level studies of the EMT phenotype

**Figure 4:**
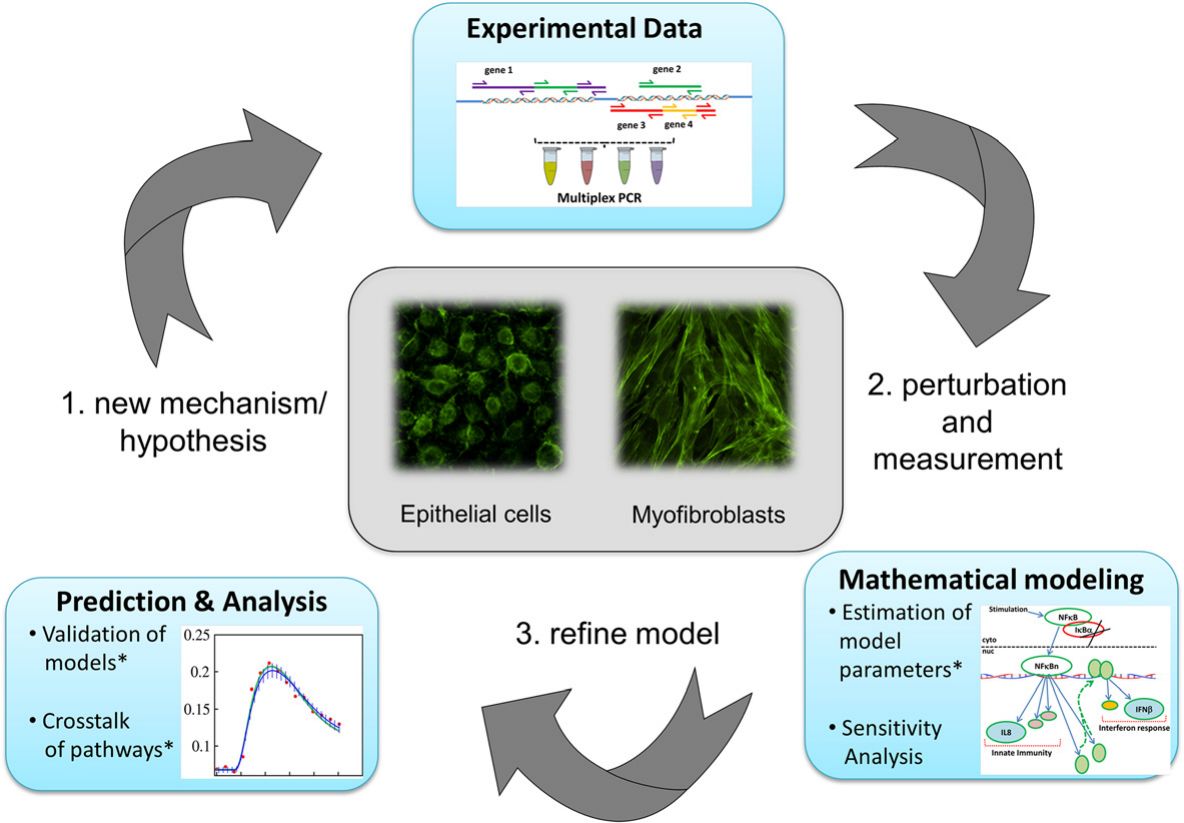
**Systems approaches to EMT.** Systems biology is an iterative process of high throughput measurement in response to perturbations, leading to refinement of predictive mathematical models, developing new mechanisms and subsequent rounds of testing.

Discussed above, the complex events produced by transcriptional reprogramming and global epigenetic rearrangement by EMT fundamentally change the inducible phenotype of the airway epithelial cell. To better understand the various asthma phenotypes, systems level studies of EMT are needed. Systems biology is an iterative process that involves the application of high throughput measurements to cellular perturbations. These data are used to develop predictive models that drive subsequent rounds of experimentation, perturbations, and predictive model refinement (Figure 4). Application of the systems biology approach can fundamentally contribute in-depth understanding of how signaling pathways and their interactions may be dysregulated by EMT (Figure 4).

A classic example of implemented framework of systems biology was the comprehensive study of the compiled gene expression data from five publicly available mouse microarray datasets and 4,305 gene annotation sets to provide the functional knowledge base [104]. A Module Networks analysis was used to identify co-regulated gene modules, inferring four distinct responses to treatment, including early response, general induction, repression, and IL-13–dependent response to the treatment. Each module consists of potential set of genes in a pathway interaction map responsible for the corresponding effect. Among the novel observations include heterogeneity in the gene expression of genetically identical animals, a transcriptionally distinct module of known and potentially novel asthma genes.

High-throughput proteomics and metabolomics have helped to classify asthmatic patients by many proteins found in the airways, which can be used clinically as useful biomarkers [105,106]. Saha *et al.* compiled the asthma biomarkers from genomics, proteomics, epigenetics and few experimentally validated datasets, and derived significant enrichment of pathways, including adipokine and ROS pathways [107]. Similarly, gene profiling studies on cancer-related type III EMT have shown that TGFβ induces global changes in mRNA expression, mRNA splicing [108] and microRNA expression [109]. Parallel proteomics studies have demonstrated that type III EMT induces growth factor independence pathways downstream of IL-6 signaling [110], which has led to a better understanding of cellular and signaling processes involved in cancer metastases [111,112].

A recent study focused on the consequences of EMT to pro-survival signaling using a correlation analysis of global measurements of protein, phosphoprotein and RNA transcript abundance [110]. These measurements were used to quantitate the differences in signaling dynamics between three different states of EMT: pre- or potential-EMT state, a reversible ‘metastable’ EMT state and an ‘epigenetically-fixed’ EMT state. The cross-correlation strategy showed differential abundance amongst multiple EMT models (218 proteins, 146 phosphoproteins and nearly 1200 RNA transcripts). A functional clustering analysis further identified significantly enriched yet differentially regulated networks between epithelial and mesenchymal states. A number of marked changes in cell-cell junctional proteins involved in cell polarity, desmosomal junctions and adhesion to basement membrane were observed with EMT. There were also substantial changes in autocrine networks (EGFR, Met and IGIFR family) and metabolic networks (redox-stress, glycolytic and oxidative pathways) with EMT. A gain of six networks with EMT including IL-11 and IL-6 mediated gp130-JAK activation was also measured. A set of EMT transcription nodes were also identified suggesting that observed differences in phenotypes could be correlated with specific transcription components, one being NF-κB2/RelA. Carefully designed systems approach revealed significant differences between epithelial and mesenchymal signaling states, thus providing new avenues for drug discovery research for modulating type III EMT. Interestingly, a genome wide profiling of chromatin signatures in human genome showed that active promoters are marked by histone-3 lysine-trimethylation whereas enhancers are marked by monomethylation [113]. Similar distinct signatures may be present and may be a predictive tool between “metastable” and “epigenetically-fixed” EMT states.

By contrast, fewer systems level studies have been applied to understand type II EMT. Although there are likely to be some important commonalities between types II and -III EMT, type II EMT is qualitatively different process because EMT of primary epithelial cells is not produced in the milieu of p53 mutation, activation of Wnt/β catenin or constitutive activation of NFκB pathways, pathways that modulate EMT. To better understand the effects of type II EMT on innate signaling, we have applied systems level studies of type II EMT using innate perturbations. Time-course perturbation experiments were used to develop parameters of mathematical models of the innate pathway [20]. Computational simulations predicted that a cap-independent translational mechanism of the rate-limiting TRAF-1-and NF-κB2 proteins was responsible for reducing the noncanonical pathway coupling interval observed experimentally. These predictions were confirmed experimentally using inhibitors of cap-independent translation [20]. In this way, application of systems modeling and perturbations provided novel insight into how the complex transcriptional reprogramming events of EMT influence dynamic response of the innate pathway.

Further applications of systems approaches will provide comprehensive understanding of this complex phenotype through deterministic modeling and multidimensional genomic and proteomic profiling (Figure 4). This kind of integrative approach can potentially provide information about cytokines and growth factors that interact to promote EMT through changes in chromatin remodeling (histone modifications, methylation), post-translational modifications (phosphoproteomics) and pathway crosstalk. Mathematical modeling of protein interactions discovered from different “omics” representing various pathways in asthma have been used to predict possible drug target genes [114]. Upon integrating data from various platforms and multiscale information from molecular to organ level, the usefulness of mathematical modeling has previously been demonstrated [115,116]. These studies provide new insights and predictions about treatment, response behavior and time, new drug formulations and optimization in a coherent manner. In addition to understanding the pathophysiology of asthma, the systems biology approach will also allow the process of developing new therapies, which will be more specific and cost-effective and yet, customized to patients phenotypes, thus expediting the clinical trials. An excellent example is the development of a novel approach for diagnosis of chronic obstructive pulmonary disease by coupling systems biology with label-free high-throughput detection [117]. Similar to the integrated proteomic studies of type III EMT discussed above, it would be interesting to see whether systems approaches can identify transcription nodes that govern type II EMT. Application of predictive biology will inform strategies for how to reprogram EMT state in complex organisms. Increasing our understanding of the role of EMT in asthma by systems biology will help us find and test personalized treatment options based upon particular characteristics of the disease phenotype.

## Translational implications

EMT is a complex, dynamic epigenetic reprogramming event that plays a homeostatic role in airway response to injury. Left unchecked, EMT contributes to pathophysiology of severe asthma. Understanding how to reverse EMT has significant translational implications. For example, EMT affects the response to glucocorticoids and inflammatory cytokines in epithelial progenitor cells, an effect that probably plays a significant pathophysiological component in a number of asthma phenotypes. It is possible that reversal of the EMT phenotype may promote allergen tolerance, enable anti-inflammatory treatment response, reduce viral-induced exacerbations, and reverse of sub-epithelial fibrosis in patients with severe asthma. The targets for reversing EMT are only beginning to be defined, and could involve targeting modulatory TGFβ signaling pathways or epigenetic modifiers. A recent investigation into possible regulators of airway remodeling found that inhibition of aldose reductase prevents TGFβ1-induced activation of the PI3K/AKT/GSK3β signaling pathways in a Smad-independent manner [115]. A separate investigation into the effects of the plant-derived propolis on TGFβ1-induced signaling pathways and EMT in alveolar epithelial cells demonstrated that this compound prevents TGFβ1-induced cellular changes, suppressed activation of Smad2 and AKT signaling pathways and may be an inhibitor of airway remodeling [116]. The central role of histone modifications and cyclin dependent kinases in EMT [20] are also worthy of further exploration.

## Summary

A hallmark of early asthma is the presence of epithelial injury/repair and expansion of subepithelial fibrosis. In the setting of chronic TGFβ stimulation from innate eosinophils and other leukocytes, modulated by ECM, epithelial cells undergo dramatic cellular transition known as type II EMT. Accumulating evidence from *in vitro*, animal and human studies suggest that chronic and refractory asthma is associated with type II EMT. EMT induces loss of mucosal barrier function, acquisition of mesenchymal characteristics and enhanced motility, and secretion of fibrotic proteins accounting for the remodeling phenotype of this disease. Here we review the evidence for EMT in producing defects in inducible phenotype of the epithelial cells including alterations in signal response characteristics of the innate response of the airways. Applications of systems level studies will be needed to unravel the dynamic and cross-talk signaling mechanisms characteristic of EMT. These exciting studies suggest that modulation of EMT may have therapeutic implications for reversing airway remodeling, restoring glucocorticoid response, and/or the innate immune defect observed in severe asthma.

## Abbreviations

ECad: E cadherin; ECM: Extracellular matrix; ECP: Eosinophilic cationic protein; EGF: Epithelial growth factor; EMT: Epithelial mesenchymal transition; EMU: Epithelial mesenchymal unit; FGF: Fibroblast growth factor; MUC: Mucin genes; NF-κB: Nuclear factor-κB; RV: Rhinovirus; SMA: Smooth muscle actin; SNAI: Snail; TGFβ: Transforming growth factor β; Th: T helper cell type; VIM: Vimentin.

## Competing interests

The authors declare that they have no competing interests.

## Authors’ contributions

All authors contributed to literature review, writing the manuscript and editing the figures. All authors have read and approved the final manuscript.
